# Synthesis, cytotoxicity and antitumour mechanism investigations of polyoxometalate doped silica nanospheres on breast cancer MCF-7 cells

**DOI:** 10.1371/journal.pone.0181018

**Published:** 2017-07-13

**Authors:** Hongqian Cao, Chunyan Li, Wen Qi, Xiangjun Meng, Rui Tian, Yanfei Qi, Wei Yang, Juan Li

**Affiliations:** 1 School of Public Health, Jilin University, Changchun, Jilin, P. R. China; 2 Department of Immunology, Norman Bethune College of Medicine, Jilin University, Changchun, P.R. China; Islamic Azad University Mashhad Branch, ISLAMIC REPUBLIC OF IRAN

## Abstract

Polyoxometalates (POMs) have shown the potential anti-bacterial, anti-viral and anti-tumor activities. In order to improve their physiological stability and antitumour activity for medical application, K_2_Na[As^**III**^Mo_6_O_21_(O_2_CCH_2_NH_3_)_3_]·6H_2_O doped silica nanospheres (POM@SiO_2_) with diameters of ~40 nm have been synthesized by the water-in-oil microemulsion method in this study. The obtained spheres were morphologically uniform nanosized and nearly monodispersed in solution. The nanoparticles had high entrapment efficiency, which was upto 46.2% by the inductively coupled plasma mass spectrometry (ICP-MS) analysis and POMs slowly released from the nanospheres both in the PH 7.4 and 5.5 phosphate buffer saline (PBS) solutions in 60 h. The *in vitro* MTT assays of particles on MCF-7 cell line (a human breast adenocarcinoma cell line) exhibited enhanced antitumor activity compared to that of plain polyoxometalate. The IC_50_ value of the POM@SiO_2_ nanoparticles was 40.0 μg/mL at 24 h calculated by the encapsulated POM concentration, which was much lower comparing to that of 2.0 × 10^4^ μg/mL according to the pure POM. And the SiO_2_ shells showed low inhibitory effect at the corresponding concentration. Confocal images further indicated the cell morphology changes and necrosis. Flow cytometric analysis showed nanoparticles induced the apoptosis by arresting the cells in S phase and western blot analysis indicated they promoted apoptosis by inhibiting the Bcl-2 protein. Moreover, the study of interactions between human serum albumin (HSA) and the nanoparticles indicated the fluorescence quenching was static, and the nanoparticles were likely to bind to HSA and changed its conformation.

## Introduction

Cancer is a serious public health problem which threatens human health in the world due to the increasing incidence and mortality. And breast cancer is one of the most common types among other malignancies in women, along with a major cause of mortality worldwide. New cases of breast cancer diagnosed in 2015 accounted for approximately 12% of all new malignancy cases and the mortality accounted for 25% of all cancer cases in women. The United Kingdom had the highest incidence among the top seven countries. Breast cancer undergoes uncontrolled growth and metastasizes to distance sites, such as brain, liver and bone [1]. The worldwide new cases of female breast cancer is estimated to reach nearly 3.2 million per year by 2050 [2]. The incidence increases with age and more than half of cases are 65 years or older [3]. In spite of treatment, >4,000 patients succumbed to the malignancy in the US in 2016 [4]. Despite intensive investigation of breast cancer cell lines, the cellular and molecular mechanisms between MCF-7 cell line and the drug polyoxometalate (POM) are still limited. In the cancer treatment, chemotherapeutic is a key method during the illness of patients. Some skin peptides obtained from amphibians even have also been demonstrated possessing the anticancer effects [5]. Because of its significance, there has been long standing interest in the development of novel approaches to improve the therapeutic index of chemotherapy.

Polyoxometalates (POMs) are outstanding class of metal-oxide clusters with O-enriched surfaces. Intriguingly, almost any other element can be incorporated into the POM frameworks, and this leads to fascinating structural versatility and rich properties [6]. It is therefore not surprising that POMs have potential applications in a variety of disciplines including catalysis [7–9], materials science [10], chemical analysis and medicine [11], etc. The previous studies have indicated that POMs are significant drug candidates owing to their remarkable antiviral, antibacterial and antitumoral activities [9, 11–17]. For example, a significant anticancer efficacy of [NH_3_Pr^i^]_6_[Mo_7_O_24_]·3H_2_O was found on MM46 adenocarcinoma and Meth A sarcoma [18, 19]. Liu and her co-workers have investigated high antitumor activity of [CoW_11_O_39_(CpTi)]_7_ on three types of cancer cells: HL-60 (leukemia), SSMC-7721 (liver cancer cell) and HLC (colon cancer cell) [20, 21]. One of the most important reasons that hinders the applicability of POMs in medicine is that many of them are thermodynamically and kinetically unstable at physiological pH and normally degrade into a mixture of inorganic products. Moreover, the excess oxo ligands of POMs lead to highly negatively charged on the surfaces and their sizes are much larger than the small nanometer sized anti-tumor molecules [17, 20–25]. The surface-charge and size characteristics reduce the penetration efficiency and the anti-tumor effect.

Surface modification of POMs with organic molecules is expected to endow the hybrid novel properties, functions or a synergetic effect. This approach has resulted in a considerable quantity of organofunctionalized derivatives, such as alkoxo, organophosphoryl, organosilyl, and organometallic derivatives [26, 27]. Experimental evaluations have already exhibited enhanced antitumour effect of POMs-based organohybrids [21]. An organic ligand offers additional advantages, such as better stability and biocompatibility. Moreover, biologically reasonable organic ligands might tune the bioactivity and cytotoxicity [28]. The encapsulation of nanoparticles with amorphous SiO_2_ shells has become a widely used technique [29, 30]. Owing to high surface area/volume ratio and relatively ease of surface functionalization, SiO_2_ shells are widely used in many fields, including the drug delivery for the controlled release of therapeutics systems [31, 32]. The silica frameworks are different from polymer nanoparticles for their immunity to enzymatic degradation and hydrolysis, as well as their large loadings of drugs [27]. Green et al. synthesized the POM/poly-L-lysine Stöber type SiO_2_ nanosphere, the conjugate led to an appealing system of drug carriers in optical monitoring field [33, 34]. The latter work reported POMs evenly spread throughout the SiO_2_ nanospheres. In general, the nanoparticles with silica coating can be achieved by the Stöber method [35] or the reverse microemulsion synthesis [36]. Human serum albumin (HSA) is a single chain polypeptide which plays a key role in maintaining normal osmolarity in plasma. It is also a main carrier for various endogenous and exogenous compounds [37, 38]. Although the antitumor activity of POMs has been investigated intensively, the binding actions between core/shell POMs decorated silica nanoparticles and HSA remain limited.

In our previous work, the *in vitro* antitumor activity of K_2_Na[As^**III**^Mo_6_O_21_ (O_2_CCH_2_NH_3_)_3_]·6H_2_O (AsMo_6_) on lung adenocarcinoma cells (A549) was investigated by the 3-[4,5-dimethylthiazol-2-yl]-2,5-diphenyl-tetrazolium bromide (MTT) assay, the results indicated that the inhibitory effect was higher than that of 5-fluorouracil (5-FU) [39]. And in the study of anti-leukemia, the compound showed low inhibitory effect on normal human umbilical vein endothelial cells (HUVEC) with an IC_50_ value of 889.18 μM and a significant anticancer effect on human HL-60 and U937 leukemia cells [40]. Herein, we aim to evaluate the cytotoxicity of pure POMs and nanoparticles on MCF-7 cell line. The K_2_Na[As^**III**^Mo_6_O_21_ (O_2_CCH_2_NH_3_)_3_]·6H_2_O doped silica nanospheres (POM@SiO_2_) was synthesized by reverse microemulsion method and confirmed by TEM and FT-IR analysis. Furthermore, their release profile was evaluated by ICP-MS. And the morphological changes of MCF-7 cells were detected by confocal laser scanning microscope. In addition, at physiological conditions, the binding interactions of the nanoparticles with HSA were investigated by multispectroscopic techniques.

## Materials and methods

### Reagents and materials

All reagents were obtained from commercial supplies and used without further purification. The K_2_Na[AsMo_6_O_21_(O_2_CCH_2_NH_3_)_3_]·6H_2_O (POM), was prepared according to the previously described approach [40]. RPMI-1640 Medium and Fetal bovine serum (FBS) were purchased from Hyclone, 3-[4,5-dimethylthiazol-2-yl]-2,5-diphenyltetrazolium bromide (MTT), phosphate buffer saline (PBS) and HSA were obtained from Sigma-Aldrich. The Propidium Iodide (PI) and Hoechst 33342 dye were obtained from Dingguo Chemicals Company. The solution of HSA was prepared by dissolving the reagent in PBS and stirred for 24 h to get a homogeneous solution. RIPA lysis buffer (Solarbio, China) and antibodies (Abcam) were prepared for the western blot analysis.

### Preparation of POM@SiO_2_ nanoparticles

The POM@SiO_2_ nanoparticles were prepared by water-in-oil microemulsion method according to the literature with a little modification [41]. A W/O microemulsion containing 9.29 mL cyclohexane, 2.26 mL n-octanol, 2.23 mL Triton X-100 and 200 μL tetraethoxysilane (TEOS) was prepared in a breaker, in the meantime, the POM (0.024g) suspended in 1 mL H_2_O was mixed with the other microemulsion containing 9.29 mL cyclohexane, 2.26 mL n-octanol, 2.23 mL Triton X-100 and 200 μL ammonia. The two microemulsions were mixed under moderate stirring at room temperature for 24 h, after that, acetone was added to precipitate the nanoparticles. The obtained white solid was washed with ethanol after centrifuging at 8000 rpm. The POM@SiO_2_ nanocomposites were dried in a desiccator under vacuum.

### Characterization of POM@SiO_2_ nanocomposites

The morphology of nanocomposites were analyzed by a transmission electron microscopy (TEM, JEM-1011) operating at an acceleration voltage of 100 kV and the POM-based particles were deposited on carbon-coated copper grids. The hydrodynamic diameter of nanoparticles was determined by dynamic light scattering (DLS) (Brookhave) analysis. The IR peaks of the nanoparticles were identified by fourier transform infrared spectroscopy (Perkin Elmer Spectrum RX-1) with the frequency range spanning from 2500 to 400 cm^−1^. The samples were prepared in the form of potassium bromide (KBr) pellets.

### Entrapment and *in vitro* release of POMs studies

The upload efficiency and *in vitro* release of POMs within the nanoparticles was determined by inductively coupled plasma mass spectrometry (ICP-MS) analysis. The *in vitro* release of POMs was performed in phosphate buffered saline (pH 5.5 and pH 7.4) at room temperature. The nanoparticles were incubated in 1.5 mL PBS buffer solution, at predetermined time intervals, the nanocomposites were centrifuged at 8000 rpm for 5 min, then 0.5 mL of the supernatant was taken out. After that, the pellet was redispersed and the same volume of PBS was supplemented. The supernatant was analyzed by the ICP-MS analysis. The entrapment efficiency was calculated by all the AsMo_6_ released from the SiO_2_ shell. It was determined by the following formulae:

$$Entrapment\;efficiency=\frac{Mnp}{Mf}\times 100\%$$ (Eq 1)

where Mnp is the mass of AsMo_6_ in the nanocomposites, Mf is the mass of POMs used in the formulation.

### Cytotoxicity studies

The breast cancer cells MCF-7 (provided by the department of the 1^st^ hospital, Jilin University) were cultured in RPMI 1640 medium (Hyclone) supplemented with 10% (V/V) fetal bovine serum (FBS, Hyclone) and 50 U/mL penicillin-streptomycin in 5% carbon dioxide atmosphere at 37°C in an incubator. The medium was changed every other day and the cells were sub-cultured after attaining about 80% confluency.

The antitumor activity of the nanoparticles was tested by the MTT assay. Briefly, The MCF-7 cells were seeded into a 96-well plate at a density of 1×10^4^ cells/well. After incubating the cells for 24 h, the dilutions of POM@SiO_2_ nanocomposites at different doses (containing real concentrations of POM 2.5–80 μg/mL at specific intervals) were added and incubated for 24 h, 48 h and 72 h. After that, the culture medium was discarded and the wells were washed with PBS twice, followed by the addition of 20 μL MTT dye (0.5 mg/mL) each well. The cells were incubated for another 4 h at 37°C. After removing all the culture medium, 150 μL DMSO was added per well. The percentage of cell viability was measured on a microplate reader (Biotek Co., USA) at the wavelength of 490 nm. The cell inhibitory rate was calculated using the following equation:

$$Inhibitory\;rate\;(\%)=(1-{OD}_{treatment}/{OD}_{control})\times 100\%$$ (Eq 2)

### Morphological observation

To observe the morphological changes on MCF-7 cells by the nanoparticles, cells were seeded in confocal culture dishes for 24 h at 37°C, after that, cells were treated with POM@SiO_2_ nanocomposites. The cells were then stained with Hoechst 33342/PI solution (1:1, V/V) at room temperature for 10 min in the dark. The cellular morphology was observed using the fluorescence confocal microscopy (Olympus FV1000, Japan).

### Flow cytometry analysis of cell apoptosis

MCF-7 cells were placed on a 12-well plate for 24 h. The nanoparticles solution with a corresponding POM concentration of 80 μg/mL was then added and incubated at 37°C. After 24 h, the cells were harvested and then washed three times with cold PBS. After centrifugation, the supernatant was discarded, and the pellets were resuspended in Annexin-V-FITC/PI buffer and left in the dark for 15 min at room temperature. Cells were analyzed on a flow cytometer using Cell Quest Software. Only single cells were gated for fluorescence analysis.

### Flow cytometry analysis of cell cycle distribution

Briefly, MCF-7 cells were seeded into a 12-well plate (1 × 10^6^ cells per well) at 37°C for 24 h. After that, they were exposed to different concentrations of the nanoparticles (corresponding POM concentrations of 80, 40 and 20 μg/mL). The cells were harvested using trypsin and collected by centrifugating at 1200 rpm, then, washed three times with cold PBS. After that, Triton X-100 (0.1%, 100 μL), DNase free RNase (200 μg/mL, 100 μL) and propidium iodide (50 μg/mL, 200 μL) were added to the cells. The cells were incubated for 30 min at room temperature in dark.

### Western blot

After treatment of POM@SiO_2_ nanoparticles for 24 h, total cellular proteins were prepared using RIPA lysis buffer from MCF-7 cells. The protein concentrations were established by bicinchoninic acid (BCA) assay. Equal amount of protein was separated by 12% polyacrylamide gels (SDS-PAGE) and then transferred onto nitrocellulose membranes. The membranes were blocked with 5% milk at 4°C overnight and then incubated with specific primary antibodies. After washing with TBST (containing 0.1% Tween 20) 3 times, the membranes were incubated with the corresponding HRP-conjugated secondary antibodies in TBST at 37°C for 1 h. The protein β-actin was used as a housekeeping control for normalization. Finally, the expression levels of proteins were visualized and analyzed using ImageJ software.

### HSA binding experiments

#### UV-vis absorption spectra

The absorption spectra of 4 mg/mL POM@SiO_2_ nanoparticles, 2.5 μM HSA and the mixture of them were measured by a UV spectrophotometer at room temperature. The absorption spectra was set from 190 to 600 nm, 10.0 mm optical path length quartz cuvette was used in this test.

#### Fluorescence titration experiments

The absorption titration experiments were performed by sequential addition of specified volume of POM@SiO_2_ nanoparticles stock solution into HSA solution. Quartz cuvette with 10.0 mm optical path length was used. Excitation of the sample took place at a wavelength of 290 nm and the emission spectra were recorded from 300 to 450 nm. The concentration of HSA was 2.5 μM, whereas the nanocomposites concentrations increased stepwise from 0 to 0.04 μg/mL. At the same time, by fixing the excitation wavelength and emission wavelength at a *Δ*λ value of 60 nm, the synchronous fluorescence intensity of the mixture solution (POM@SiO_2_ nanoparticles and HSA) was detected. The widths of the slits were all 5.0 nm.

### Statistical analysis

All statistical analyses were performed using the SPSS Version 17.0 for Windows (significance was established at *P* < 0.05). Data were expressed as mean ± SD (standard deviation), statistical significance was evaluated by one-way analysis of variance (ANOVA) combined with Duncan’s multiple range tests. All experiments were performed in triplicate, unless otherwise indicated.

## Results and discussions

### Synthesis and characterization of the POM@SiO_2_ nanoparticles

**Fig 1:**
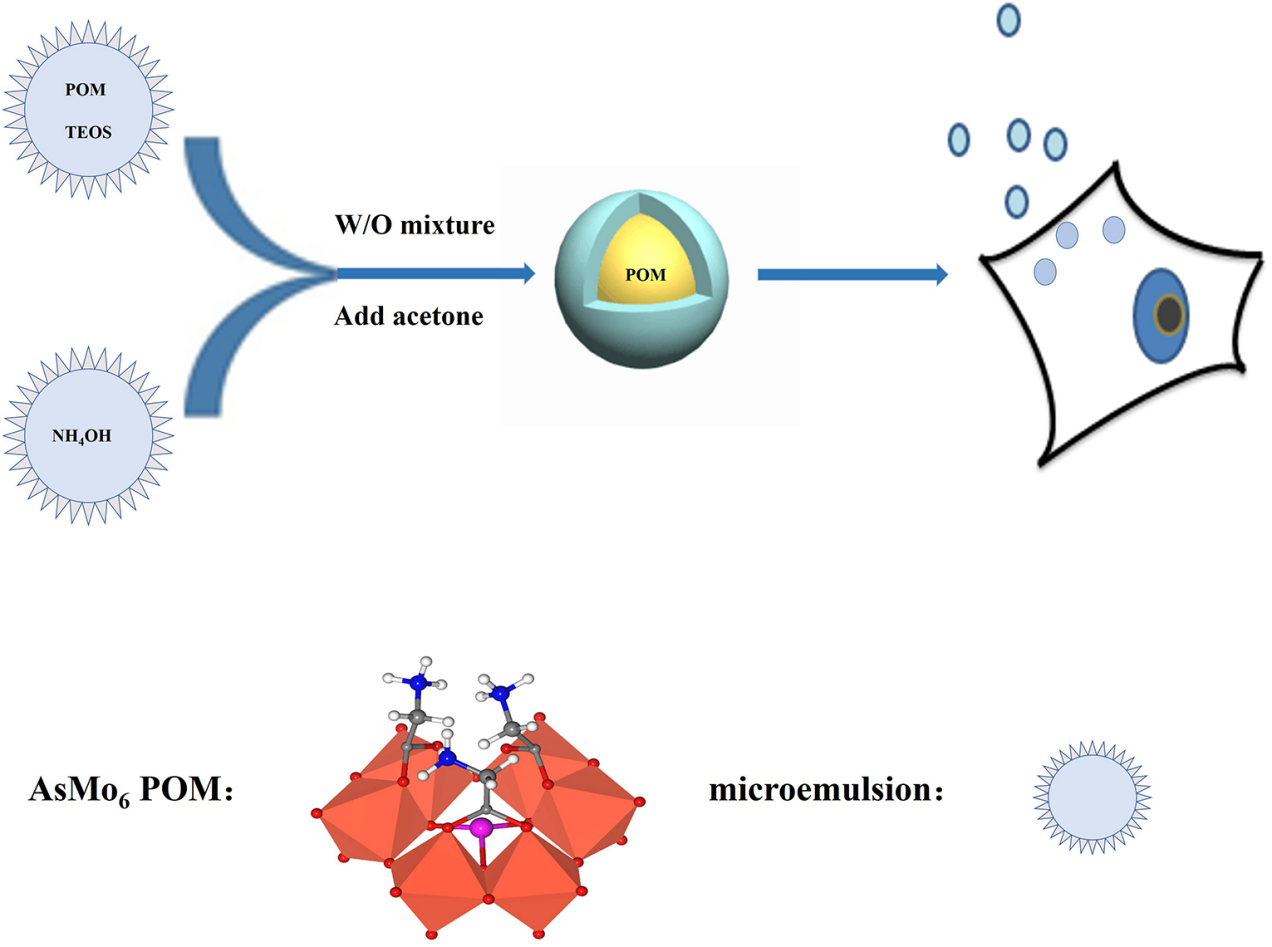
Schematic of the synthesis of POM@SiO_2_ nanoparticles using in the antitumour procedure.

**Fig 2:**
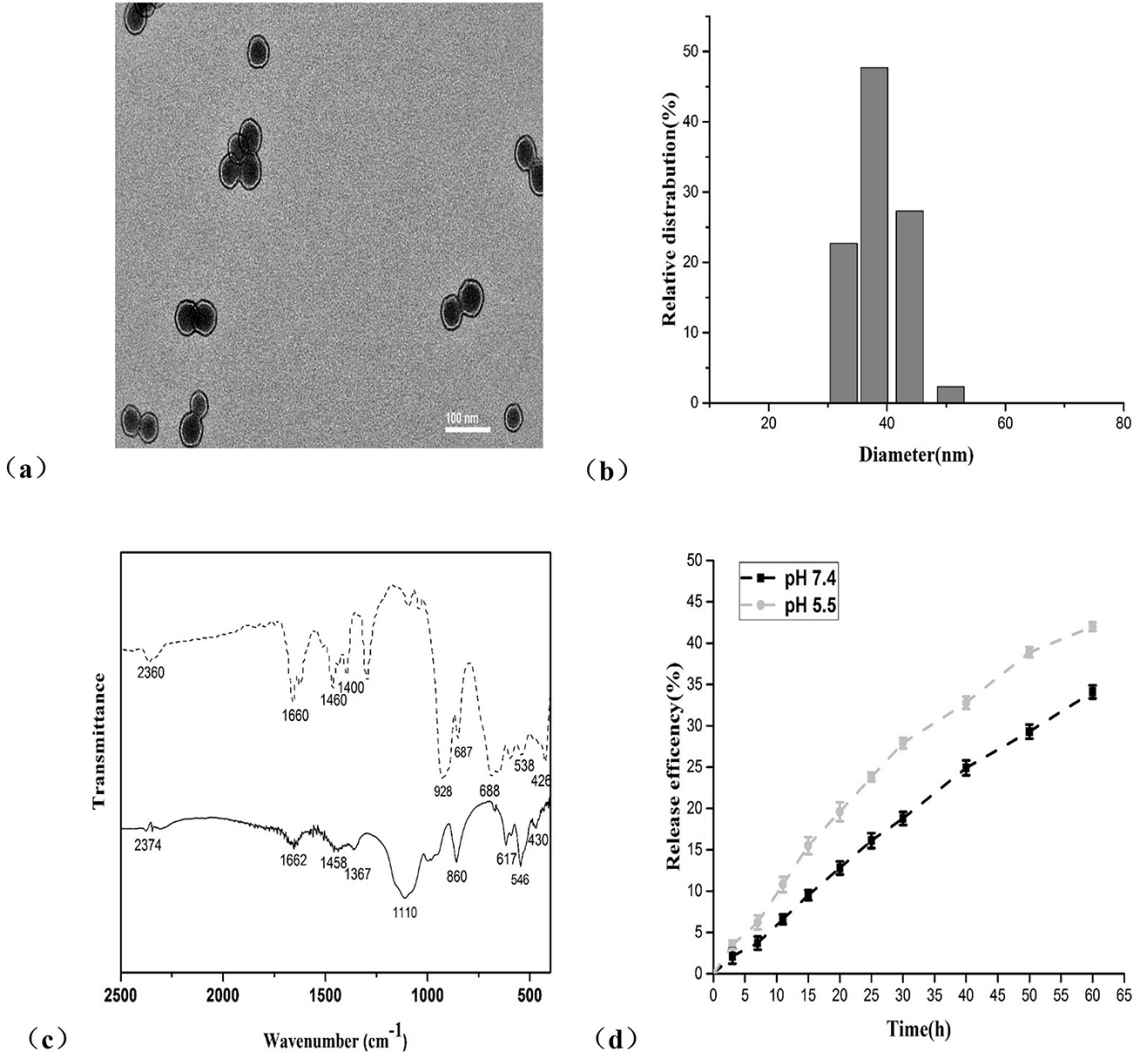
Characterization and release efficiency of the POM@SiO_2_ nanoparticles. **(a)** TEM images of AsMo_6_@SiO_2_ nanoparticles. The scale bar is 100 nm. **(b)** DLS measurement image of the nanoparticles. **(c)** FT-IR spectra of AsMo_6_ (dash line) and nanoparticles (solid line). **(d)** The release efficiency of nanoparticles at pH 7.4 and 5.5. Results represent the mean ± SD from three independent experiments.

The POM@SiO_2_ nanoparticles were obtained by the hydrolysis of tetraethoxysilane (TEOS) method (Fig 1). The TEM image showed morphological uniform nanosized spheres (Fig 2A), which provided direct evidence of the formation. It is clearly evident from the image that nearly monodispersed POM@SiO_2_ nanoparticles were obtained by this technique, the average particle diameters were about 39 nm. The POMs were encapsulated in SiO_2_ with an evident core/shell structure. EDX mapping confirmed the presence of arsenic and molybdenum from POM and the presence of silica from the shell (S1 Fig). As shown in Fig 2B that the hydrodynamic diameter of POM@SiO_2_ nanoparticle solution was between 30 and 52 nm by the dynamic light scattering (DLS) measurements. The image was supported by the above transmission electron microscopy (TEM) observation. Particles between 33 and 43 nm reached about 48%. These results also demonstrated the reduction of POM aggregation due to the encapsulation. The FT-IR spectra (Fig 2C) of the POM exhibited the absorption characteristic peaks at 928, 688, 538 and 426 cm^-1^, which were attributed to *ν*(Mo-O_bridge_-Mo) and *ν*(Mo-O_terminal_) of polyoxoanion as described in the previous study [39], the corresponding peaks were exhibited in the POM@SiO_2_ solid line as shown in the image (860, 617, 546, 430 cm^-1^), indicating that these compounds still retain the basic structure after encapsulated in SiO_2_. The strong absorption band at 1110 cm^-1^ as shown in the POM@SiO_2_ line was attributed to the Si-O stretching, which further demonstrated the formation of POM@SiO_2_ nanoparticles.

To characterize the release of POMs, the POM@SiO_2_ nanoparticles were incubated at pH values of 5.5 and 7.4 PBS solutions. The nanoparticles were centrifuged at predetermined time intervals and analyzed by ICP-MS. From these releasing profile studies (Fig 2D), the AsMo_6_ was slowly released from the nanoparticles at every detecting point. At the point of 25 h, 16.1% was released from the particles in pH 7.4 PBS and 23.8% in pH 5.5 PBS. At the point of 60 h, reached about 34% and 42%, respectively. The release efficiency increased stepwise during the intervals and the SiO_2_ resulting in the controlled release from the shells. Efficiency at pH 5.5 was a little higher than that at pH 7.4. Entrapment efficiency was calculated by all the POMs which released from the nanocomposites. The entrapment efficiency of POM within the nanoparticles was calculated to be 42.6%.

### Anticancer activity studies

**Fig 3:**
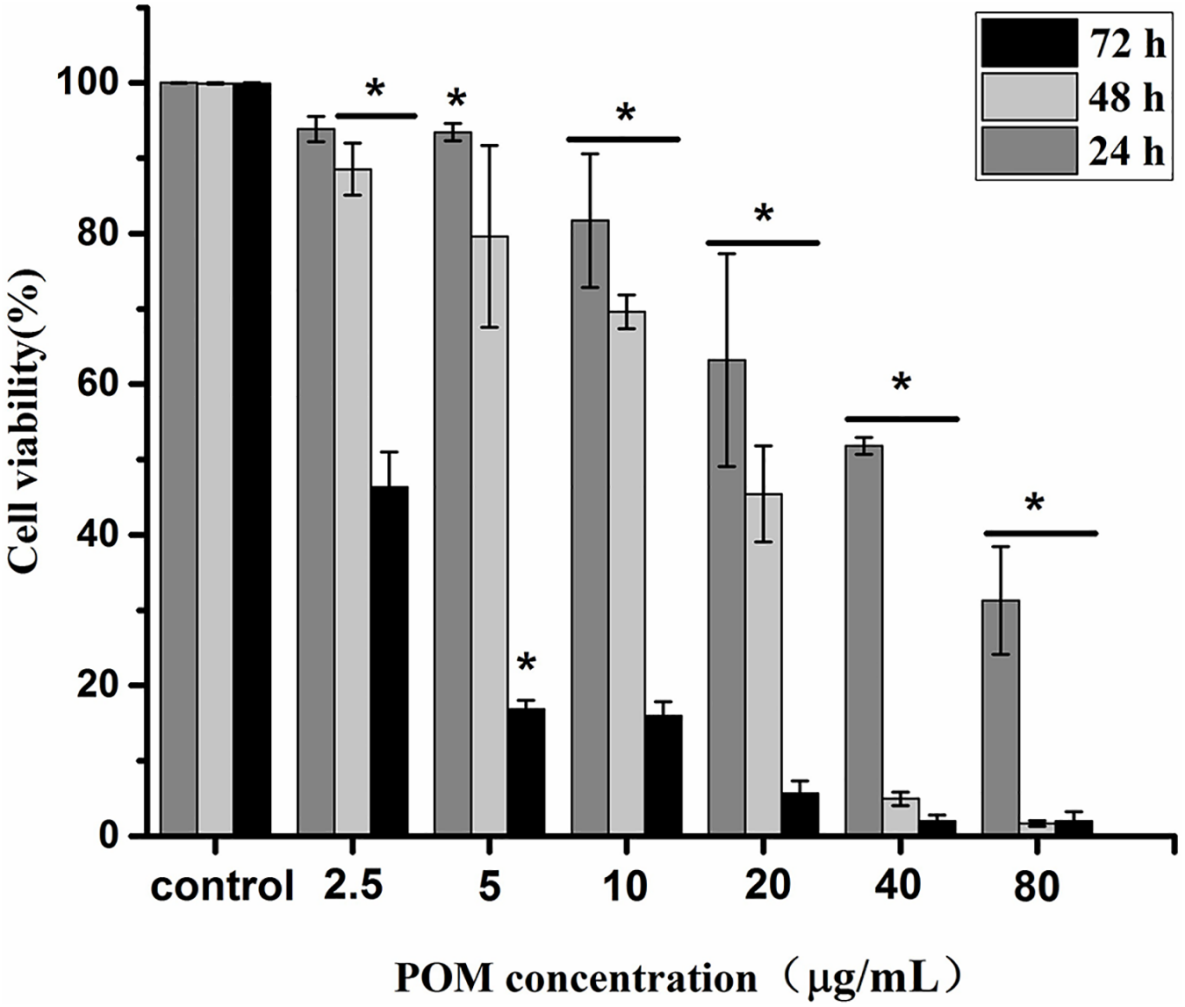
Nanoparticles show antitumor activity in a time and dose dependent manner. Cytotoxicity analysis of POM@SiO_2_ nanoparticles on MCF-7 cells by MTT at different doses. The POM concentration was calculated by the encapsulate efficency of the nanoparticles. Data are presented as the mean ± SD of three independent experiments. *P < 0.05 for the nanoparticles at different doses *vs*. control.

To determine whether the doped POMs nanoparticles could enhance their antitumour activity, experiments were performed *in vitro* at different concentrations by MTT assay on MCF-7 cells. As shown in Fig 3, the nanoparticles showed significant cytotoxicity to MCF-7 cells at a concentration of 40 μg/mL (calculated by the encapsulated POM) with the cell viability about 51.9%, the anti-proliferative effects were dependent on its concentration. The cell viability was down to 31.3% at 24 h, 1.7% at 48 h and still kept about 2.0% at 72 h with POM concentration of 80 μg/mL that contained in the nanoparticles. The IC_50_ value of the corresponding POM concentration in the POM@SiO_2_ nanoparticles was calculated to be 40 μg/mL at 24 h, 10.8 μg/mL at 48 h, 1.7 μg/mL at 72 h. Particles showed antitumor activity in a time dependent manner in these three periods. For one reason, the cells took more particles and the drug exhibited more antitumor effect with longer time during a specific time frame, which could enhance the cytotoxicity. For the other, the SiO_2_ shells encapsulated the drug inside, which could control and decrease the release efficiency of POM as mentioned in the drug release experiment. At the point of 48 h, the release efficiency was about 37% at pH 5.5 and the drug showed significant antitumor activity to cells at the biggest drug concentration. In this way, the nanoparticles showed enhanced cytotoxicity during the long period as detected at different intervals. On the other hand, as shown in S2A Fig, the pure POM solution with the same concentration that contained in the particles exhibited nearly no cytotoxicity on MCF-7 cells. The IC_50_ value of the plain drug was calculated to be 2.0 × 10^4^ μg/mL (S2B Fig), which was much larger than that according to the nanoparticles. At the concentration of 1.8 × 10^4^ μg/mL, the cell viability was 33.6%. For the SiO_2_ shells account for most mass of the particles, the cytotoxicity of the corresponding concentration shells was performed. The SiO_2_ at the same concentration which performed in the cytotoxicity study of the nanoparticles showed little antitumor effect (S3 Fig). The cell viability was 79.9%, 69.5%, 66.5% comparing to that of 63.2%, 51.8% and 31.3% as shown in results of the nanoparticles. It can be concluded that the antitumor activity of the POM@SiO_2_ nanoparticles is higher than the free POM_._

### Hoechst 33342/PI double staining assay

**Fig 4:**
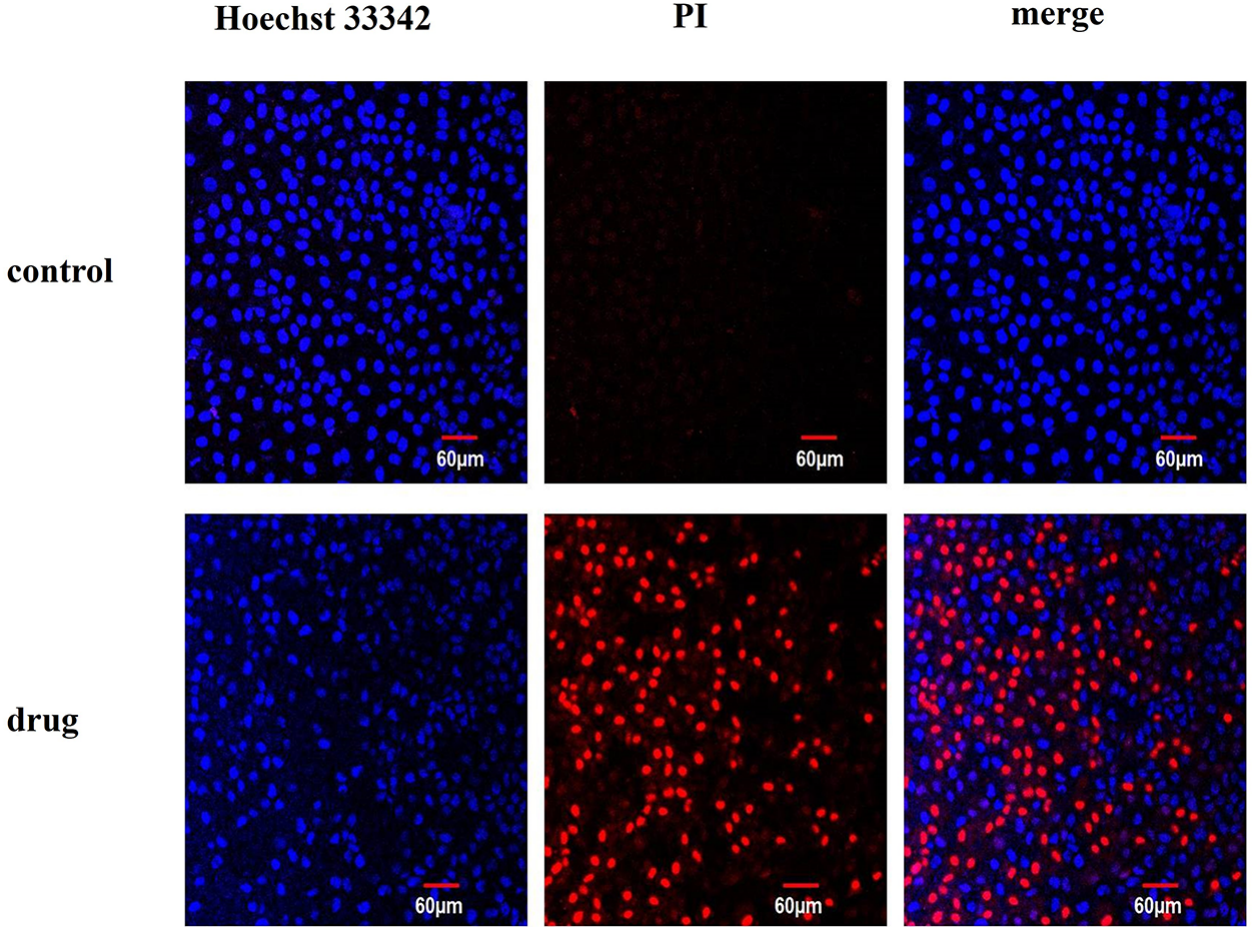
Hoechst 33342/PI dye shows the normal and dead cells. MCF-7 cells treated with POM@SiO_2_ nanoparticles (corresponding POM concentration of 80 μg/mL) at 24 h and stained with Hoechst 33342/PI dye. Scale bar is 60 μm (Original magnification 20 ×).

Hoechst 33342/PI double staining assay was performed to detect the necrotic cell by confocal laser scanning microscopy (Fig 4). The nuclei of normal cells can be stained into light blue (Hoechst 33342), apoptotic cells can be stained into brilliant blue and light red (Hoechst 33342 and PI) and the dead cells were brilliant red (PI). The results were presented in the CLSM images. In the control group, nuclei were all stained into blue and no red cells appeared in the image. In the presence of POM@SiO_2_ nanoparticles, the cell numbers reduced dramatically and there were a large number of necrotic cell residues (stained into red by PI). Massive necrotic and apoptotic cells were found in the drug group at the corresponding POM concentration of 40 μg/mL. The number of dead cells were increased with the adding of nanoparticles. Therefore, it can be concluded that the nanoparticles inhibit this cell line growth significantly. The changes of nuclei and cells morphology become much clearer under the magnification of 60 (S4 Fig). Cells in the control groups nested very well and grew strongly at a high density (bright images of DIC channel). Comparing to the intact and polygonal shape in the control group, cells exposed to the nanocomposites showed shrink margin loss of contact with the adjacent ones and apparently the sharp decrease in amount. Hoechst 33342/PI cytological investigations elucidated the membrane instability and cytoskeleton disturb effect by the nanoparticles.

### Flow cytometry analysis of cell apoptosis

**Fig 5:**
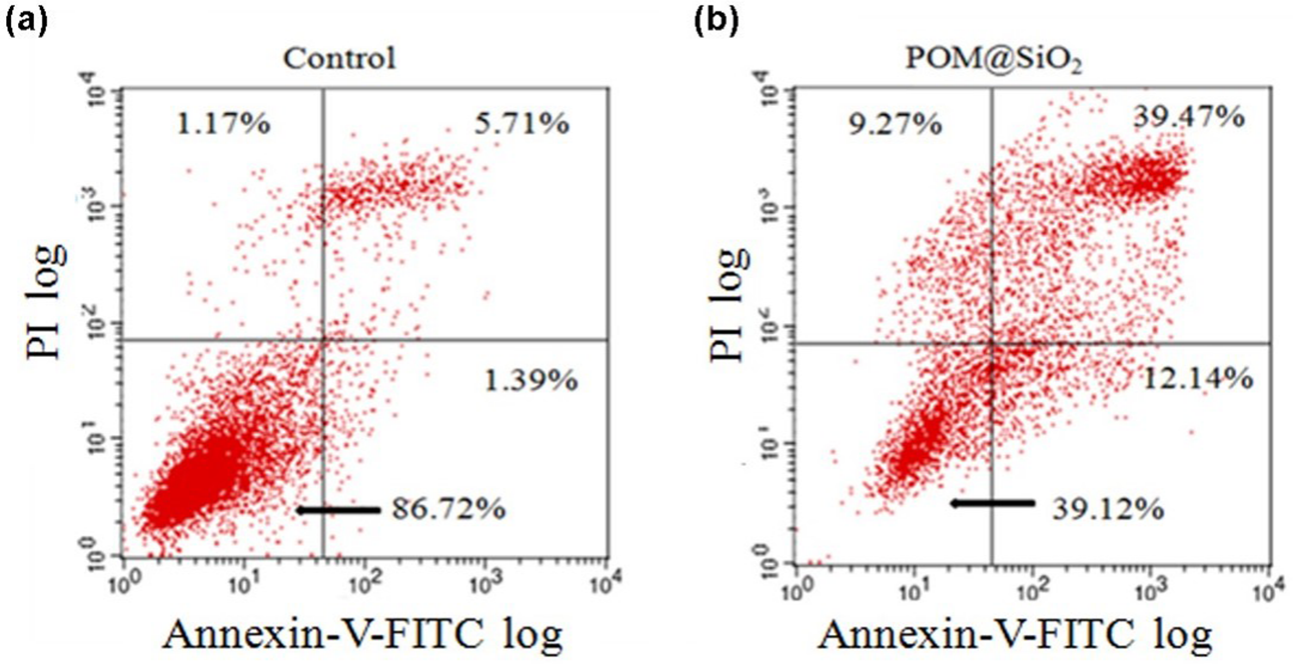
Annexin V-FITC/PI analysis of apoptosis in MCF-7 cancer cells induced by POM@SiO_2_ nanoparticles. The corresponding POM concentration is 80 μg/mL. Cells were incubated with nanoparticles for 24 h. The lower-right panel presents early apoptotic cells, whereas the upper-right panel displays late apoptotic cells. **(a)**The control group. **(b)** The POM@ SiO_2_ treated group.

To further confirm and detect the apoptosis effect on MCF-7 cells induced by the nanoparticles, Annexin V-FITC /propidium iodide (PI) double-staining technique was used. It is sensitive to detect the early apoptosis phase cells based on distinct double staining patterns: viable (Annexin V- and PI-, lower left square), early apoptotic (Annexin V+ and PI-, lower right square), late apoptotic (Annexin V+ and PI+, upper right square) and necrotic cells (Annexin V- and PI+, upper left square). The results (Fig 5) showed that the proportion of late apoptotic cells increased with the addition of the nanoparticles. The proportion of late apoptotic cells induced by the nanoparticles is nearly 34% higher than the control group (5.71% in control group, 39.47% in drug group). Taken together, the POM@SiO_2_ nanoparticles induced the apoptosis of MCF-7 cells and inhibited their proliferation, which were consistent with the MTT results mentioned above.

### Flow cytometric analysis for cell cycle distribution

**Fig 6:**
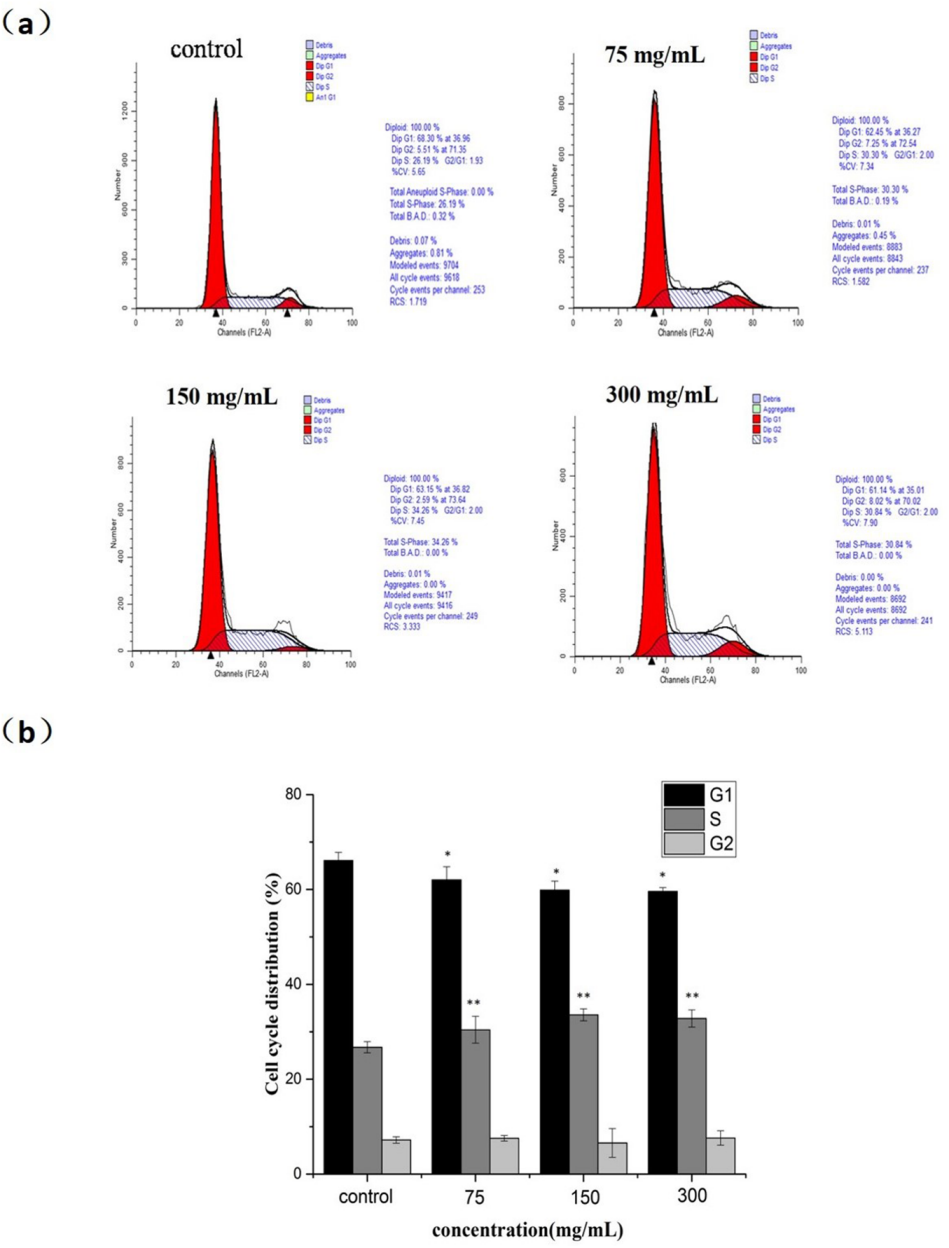
Cell cycle arrest effect of nanoparticles on MCF-7 cells. **(a)** Cells were treated with POM@SiO_2_ nanoparticles (corresponding POM concentration of 20, 40, 80 μg/mL) for 24 h. The distribution of cell cycle was detected by FCM with PI staining. The percentages of G_1_, S, G_2_ phase were calculated using ModFit LT software. **(b)** Statistical results of S phase cells. Results represent the mean ± SD from three independent experiments. *P < 0.05 for G_1_ phase *vs*. control. **P < 0.01 for S phase *vs*. control.

To detect whether the growth inhibition of MCF-7 cells by nanoparticles is a result of cell cycle arrest, MCF-7 cells were seeded and exposed to different concentrations of the POM@SiO_2_ nanoparticles (corresponding POM concentrations of 20, 40, 80 μg/mL) for 24 h, stained with PI and examined by FCM to analyze the changes of cell cycle. Generally, a doubling of DNA and other cellular contents are necessary for a cell replication process. Cell cycle distribution is divided into four distinct phases: G_1_ phase, S phase, G_2_ phase and M phase, which are defined by cell entry checkpoints. The major event of S phase is the synthesis of DNA. The cell prepares to divide during G_2_ phase and division takes place during M phase. Passing these checkpoints, the cell will proceed with division. External factors such as drugs (5-fluorouracil), radiation, and reactive oxygen species result in DNA-damage-related cell death during S phase [42, 43]. The abnormality of DNA prevents the progress of replication and influences the next new one. Then the DNA repair occurs during the G_2_ phase, before entering the M phase for mitosis. For the cell cycle analysis, as shown in Fig 6, the results showed increase level in S phase from 26.73% in the control group to 30.41%, 33.58%, 32.81%, respectively with the corresponding reduction in G_1_ and G_2_/M phase. The level of G_1_ phase was 66.1%, 62.04%, 59.86% and 59.58%, respectively, which suggested the inhibition mechanism on MCF-7 cells was S phase arrest. Since DNA replication occurs during S phase, DNA damage is responsible for S phase arrest as well as the inhibition of proliferation versus the control group.

### Western blot

**Fig 7:**
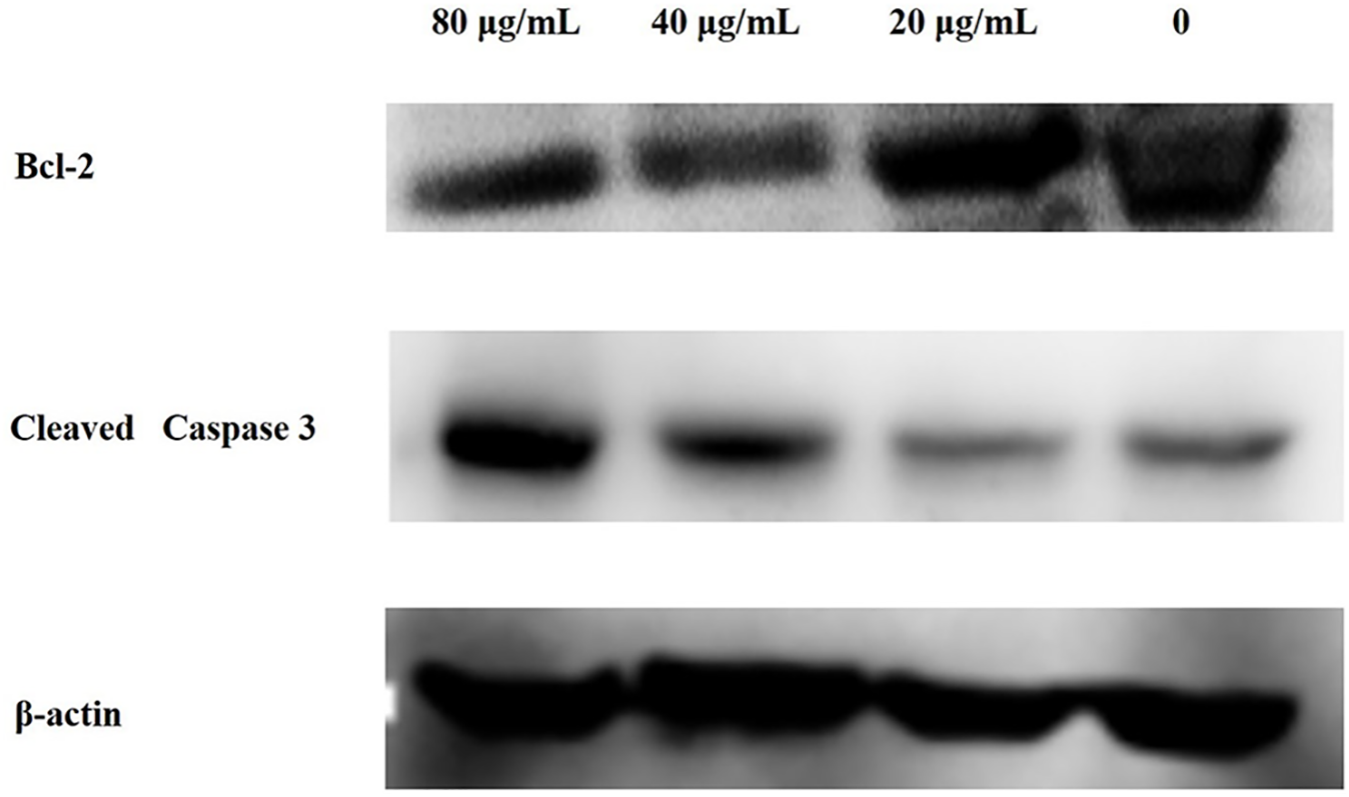
Effect of POM@SiO_2_ on the expressions of β-actin, Bcl-2, and cleaved caspase 3 proteins. The MCF-7 cells were treated with different concentrations of POM@SiO_2_ nanoparticles (corresponding POM concentration of 20, 40, 80 μg/mL) for 24 h. The nanoparticles induced apoptosis by inhibiting the Bcl-2 protein.

The flow cytometry analysis indicated that the apoptosis rate increased in MCF-7 cells as the POM@SiO_2_ nanoparticles concentration increased. To further confirm the effects of nanoparticles in MCF-7 cells, western blot assay was performed to investigate the molecular mechanism for the observed apoptosis. As shown in Fig 7, a remarkable upregulation of cleaved caspase 3 protein levels and a decrease of Bcl-2 protein levels were observed as the nanoparticle concentration increased. As we know Bcl-2 is an apoptosis inhibitor which could restrain cell apoptosis. The cleaved caspase 3/β-actin ratio in MCF-7 cells treated with nanoparticles (corresponding POM concentration of 80 μg/mL) was 0.616, which was significantly higher than that of the blank control. Therefore, our findings showed that nanoparticles could promote apoptosis by inhibiting the Bcl-2 protein and upregulating the cleaved caspase 3 expression in cells.

### HSA binding

#### UV-vis absorption

**Fig 8:**
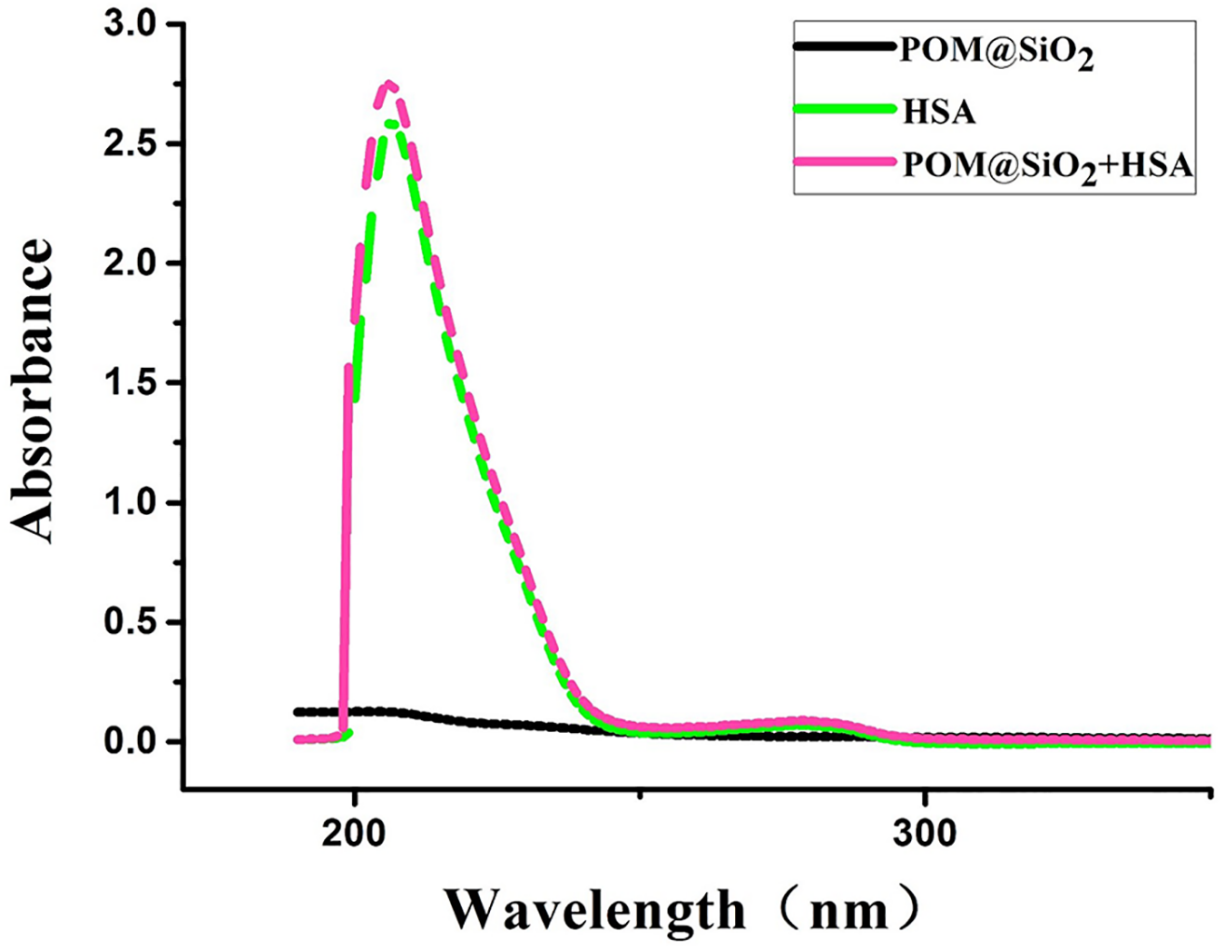
Effect of nanocomposites on the absorption spectra of HSA. **(a)** UV-vis spectra of 2.5 μM HSA, **(b)** POM@SiO_2_ nanoparticles (4 mg/mL) and **(c)** nanoparticles-HSA complex.

UV-vis measurement is an easy and effective way to investigate the structural changes and complex formation between the protein and nanoparticles [44]. As shown in Fig 8, the characteristic absorption peaks of isolate HSA were 206 nm and 278 nm, meanwhile isolate POM@SiO_2_ nanoparticles were found near 200 nm at the same concentration. With the addition of nanoparticles solution, the absorption peak of the protein was slightly increased, which indicated that an interaction occurred between HSA and POM@SiO_2_ nanoparticles.

### Fluorescence spectroscopic experiments

The fluorescence measurements can be applied to investigate the binding information of small molecule substances to protein, such as the binding mechanism, binding constants, binding mode, and the number of binding sites. Fluorescence quenching is the decrease of quantum yield from a fluorophore induced by quencher molecule, there are variety molecular interactions between them, such as excited-state reaction, energy transfer and collision quenching. That means the fluorescence changes can give information about the molecular micro-environment. And in another level, changes in emission spectra of residues is often considered in the investigation of protein folding and association reactions [5, 45]. Fluorescence quenching is a well-established method to study the interactions between ligand and protein. It has been used to analyze the interactions between different POMs and HSA [46–50]. But all of these investigations were previously performed between plain POMs and protein. In this study, the interaction between POM uploading silica nanoparticles and HSA was performed.

**Fig 9:**
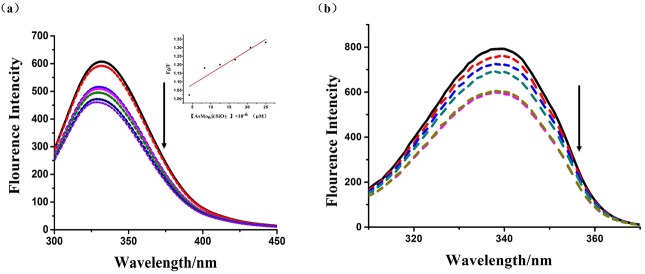
Fluorescence quenching and binding effect study of the nanocomposites. **(a)** Fluorescence spectra of HSA (2.5 μM) in the presence of different concentrations of nanoparticles. The arrow shows the fluorescence intensity changes upon increasing concentration of nanoparticles. Inset: Stern-Volmer plot of the fluorescence titration data of HSA with different concentrations of nanocomposites in PBS at room temperature ([POM@SiO_2_] = 0, 0.125, 0.25, 0.5, 1, 2, 4 × 10^−2^ μg/mL). **(b)** The synchronous fluorescence spectra of HSA (0.02503B0043M) in the presence of different concentrations of nanoparticles at Δλ = 60 nm.

HSA belongs to endogenous fluorophores with tryptophan residue at position 214 nm in the molecule. In these steady state fluorescence experiments, the concentration of HSA was kept constant, while the concentration of nanoparticles was increased stepwise. The fluorescence emission spectra of the complexes in the absence and presence of HSA were shown in Fig 9A. [51]. Fluorescence intensity of HSA decreased regularly at about 330 nm with the enhancement of nanoparticles (fluorescence quenching), which indicated that particle interacted with HSA and a complex is formed between them [45]. Furthermore, there was a red shift at the maximum emission peaks of HSA after the addition of particles, which suggested that the fluorescing residues in nonpolar hydrophobic cavities are moved to a hydrophilic environment [5].

In the case of fluorescence quenching procedures, the absorption of nanoparticles can absorb protein fluorescence and the presence of particles can scatter the input and output fluorescence, further reducing the fluorescence signal, which are termed as inner filter effect (IFE). This also results in a decreased fluorescence intensity that is not a quenching effect. In this study, the following formula was used for correcting fluorescence intensity:

$${F_{cor}=F_{obs}\times 10}^{(Aex+Aem)/2}$$ (Eq 3)

where F_cor_ and F_obs_ are the correct and observed fluorescence intensity, respectively. A_ex_ and A_em_ are the absorbance value at the excitation and emission wavelengths, respectively. Fluorescence intensities were corrected for inner filter and dilution effects before the following analysis [45, 52].

There are two possible reasons for the complexes quenching HSA fluorescence, the static quenching and dynamic quenching. The analysis of the results was done with the help of Stern-Volmer equation [53]:

$$F_{0}/F=1+K_{q}\tau_{0}[D]=1+K_{sv}\;[D]$$ (Eq 4)

where F_0_ and F are the fluorescence intensity in the absence and presence of a quencher, respectively, [D] is the concentration of quencher, K_q_ and K_sv_ are the quenching rate constant, Stern-Volmer dynamic quenching constant, respectively, τ_0_ is the average lifetime of molecule without the quencher and its value is 10^−8^ s. Based on the experimental data, the Stern–Volmer plot for the quenching of HSA with POM@SiO_2_ nanoparticles was given in the inset of Fig 8A. If the quenching process is the dynamic one, the dependence of F_0_/F on the quencher concentration is linear with slope equal to the value of K_sv_. Moreover, the maximum quenching rate constant of various quenchers is 2.0 × 10^10^ Lmol^-1^s^-1^. The fluorescence quenching coefficient of the POM@SiO_2_ is 1.3 × 10^18^ Lmol^-1^s^-1^, which is far greater than the K_q_ value of the dynamic quenching for various quenchers. It suggested that the quenching belonged to a static quenching rather than the dynamic one. In static quenching, small molecules bind to equivalent sites on a macromolecule independently; whereas in the dynamic interaction, the quencher binds to the fluorophore during the excited state [5]. Furthermore, static quenching often changes absorption spectrum of the luminophore molecule, while dynamic quenching is on the contrary. According to the UV-vis analyses, the absorption spectrum of HSA was changed by the POM@SiO_2_. Therefore, the fluorescence quenching of the nanoparticles was static quenching and the nanoparticles were likely to bind to HSA. These results indicated the interaction between POM@SiO_2_ nanoparticles and HSA molecules, and the nanospheres can be transported in the serum albumin.

To further study the binding effect of nanoparticles to HSA, synchronous fluorescence (SFS) study was performed. The test could provide the information of tryptophan residue of the protein when the wavelength interval was established in 60 nm [33]. Fig 9B showed that the fluorescence intensities of tryptophan residue were decreased as the nanoparticle solutions added stepwise to HSA stock solution. The emission wavelength peak of tryptophan residue in the investigated concentration range was a slightly blue shift (Δλ = 60 nm). It suggested that the nanoparticles changed the conformation of the protein.

## Conclusions

In summary, we report the *in vitro* anti-proliferative effects on MCF-7 cells of the POM@SiO_2_ nanoparticles with small size around 40 nm. The nanoparticles significantly enhanced the cytotoxicity on the cells comparing to that of the pure POM. SiO_2_ shells controlled the release efficiency as the drug delivery system. The higher antitumor activity could be due to their higher cellular penetration for the small size and surface modification by the shells. The Flow cytometry analysis results showed that the nanoparticles could induce apoptosis and cell cycle S phase arrest. The HSA binding experiment showed that the complex changed the conformation and quenched the fluorescence of the protein, which belonged to a static quenching. Furthermore, the antitumour effect of other cell lines and modification with target molecules on the spheres should be further investigated.

## Supporting information

S1 Fig EDX mapping images of POM@SiO_2_ nanocomposites. TEM image in dark field mode **(a)** and EDX mapping for Si (green) **(b)**, As (red) **(c)** and Mo (blue) **(d)**. The scale bar is 50 nm.
(TIF)

S2 Fig The inhibitory effect of plain POM solution on MCF-7 cells at 24 h. **(a)** Cell viability of the corresponding concentration performed in the cytotoxicity investigation of the nanoparticles. **(b)** Cell viability of higher concentration of plain POM. Results represent the mean ± SD from three independent experiments. *P<0.05 for POM *vs*. control.
(TIF)

S3 Fig The inhibitory effect of pure SiO_2_ MCF-7 cells at 24 h. The SiO_2_ at the same concentration performed in the cytotoxicity study of the nanoparticles showed low antitumor effect.
(TIF)

S4 Fig Morphological changes of MCF-7 cells treated with POM@SiO_2_ nanoparticles at 24 h. The corresponding POM concentration was 80 μg/mL. Scale bar in the CLSM is 40 μm (Original magnification 60×). Arrow shows apoptotic nuclear.
(TIF)
