# Supplementary figures and images for: Synthesis, cytotoxicity and antitumour mechanism investigations of polyoxometalate doped silica nanospheres on breast cancer MCF-7 cells
Source: PLoS One. 2017 Jul 13;12(7):e0181018. doi: 10.1371/journal.pone.0181018 (PMC5509251; doi:10.1371/journal.pone.0181018)

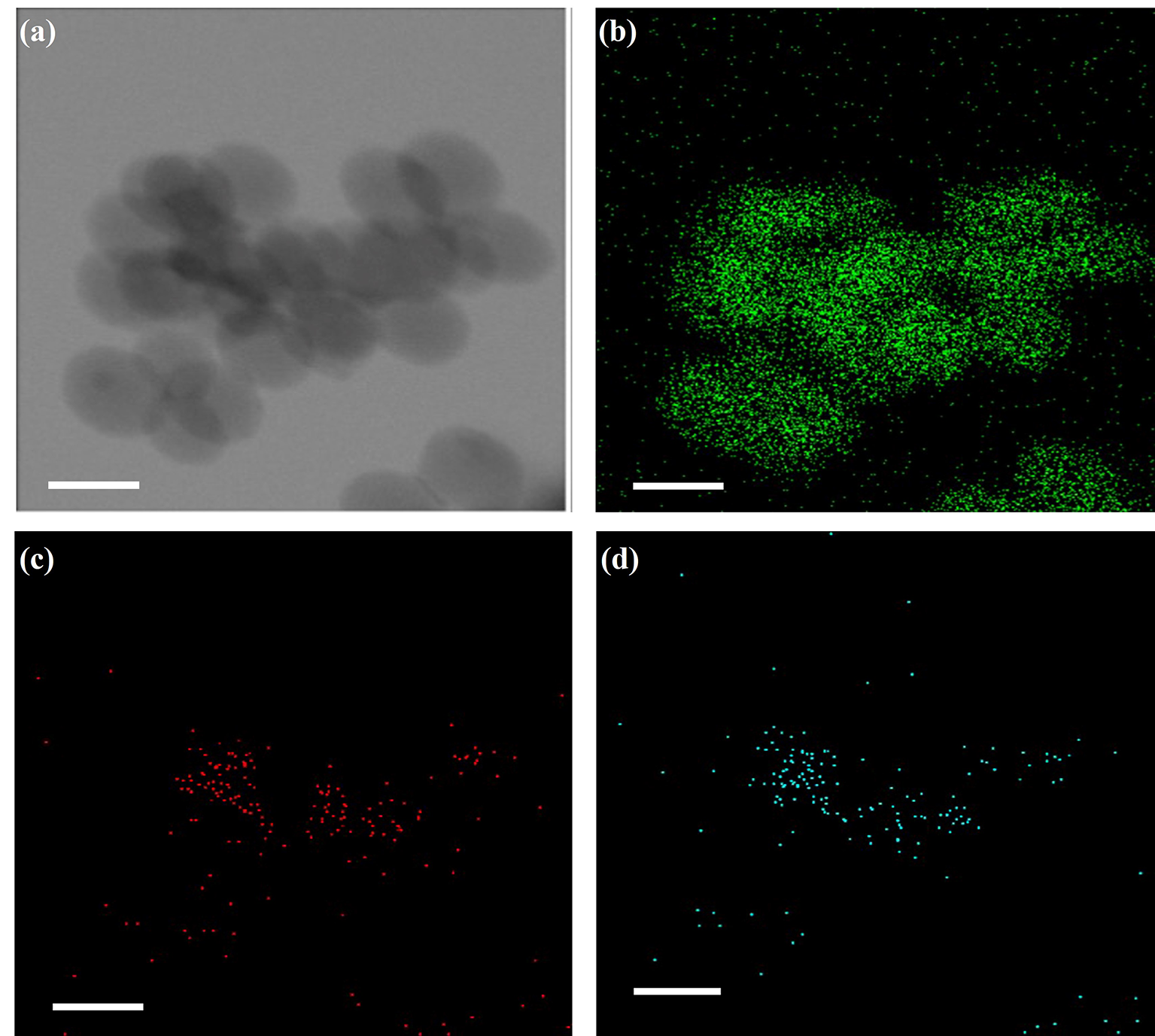

Supplement: S1 Fig — TEM image in dark field mode (a) and EDX mapping for Si (green) (b), As (red) (c) and Mo (blue) (d). The scale bar is 50 nm. (TIF) [file pone.0181018.s001.tif]

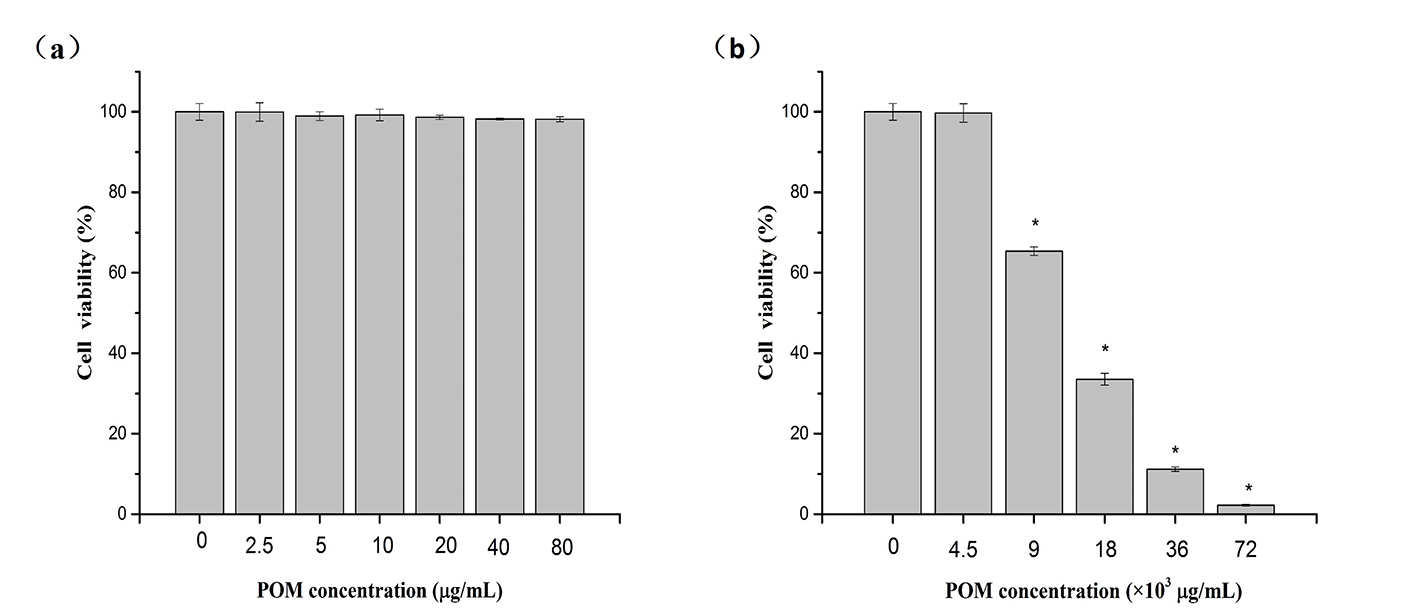

Supplement: S2 Fig — (a) Cell viability of the corresponding concentration performed in the cytotoxicity investigation of the nanoparticles. (b) Cell viability of higher concentration of plain POM. Results represent the mean ± SD from three independent experiments. *P<0.05 for POM vs. control. (TIF) [file pone.0181018.s002.tif]

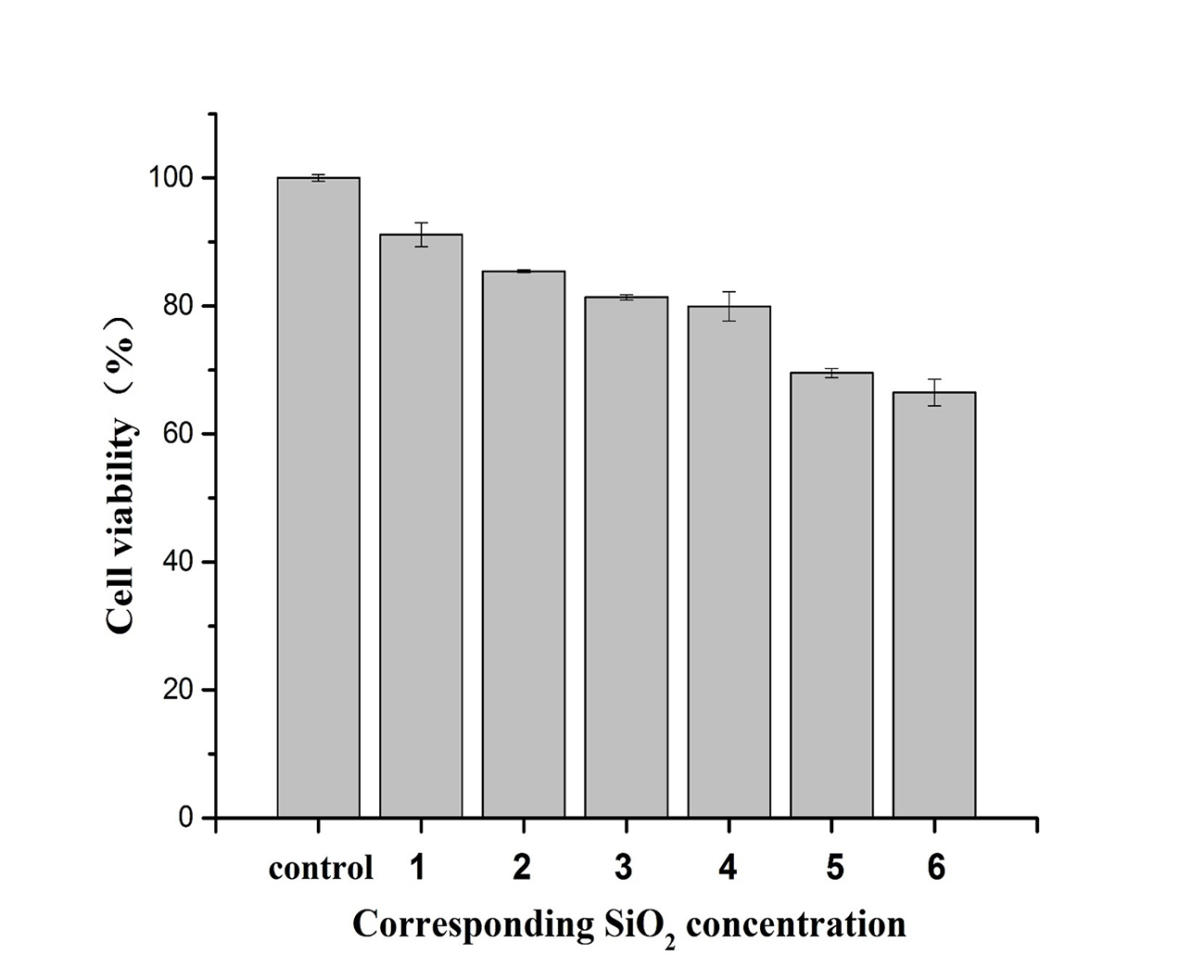

Supplement: S3 Fig — The SiO2 at the same concentration performed in the cytotoxicity study of the nanoparticles showed low antitumor effect. (TIF) [file pone.0181018.s003.tif]

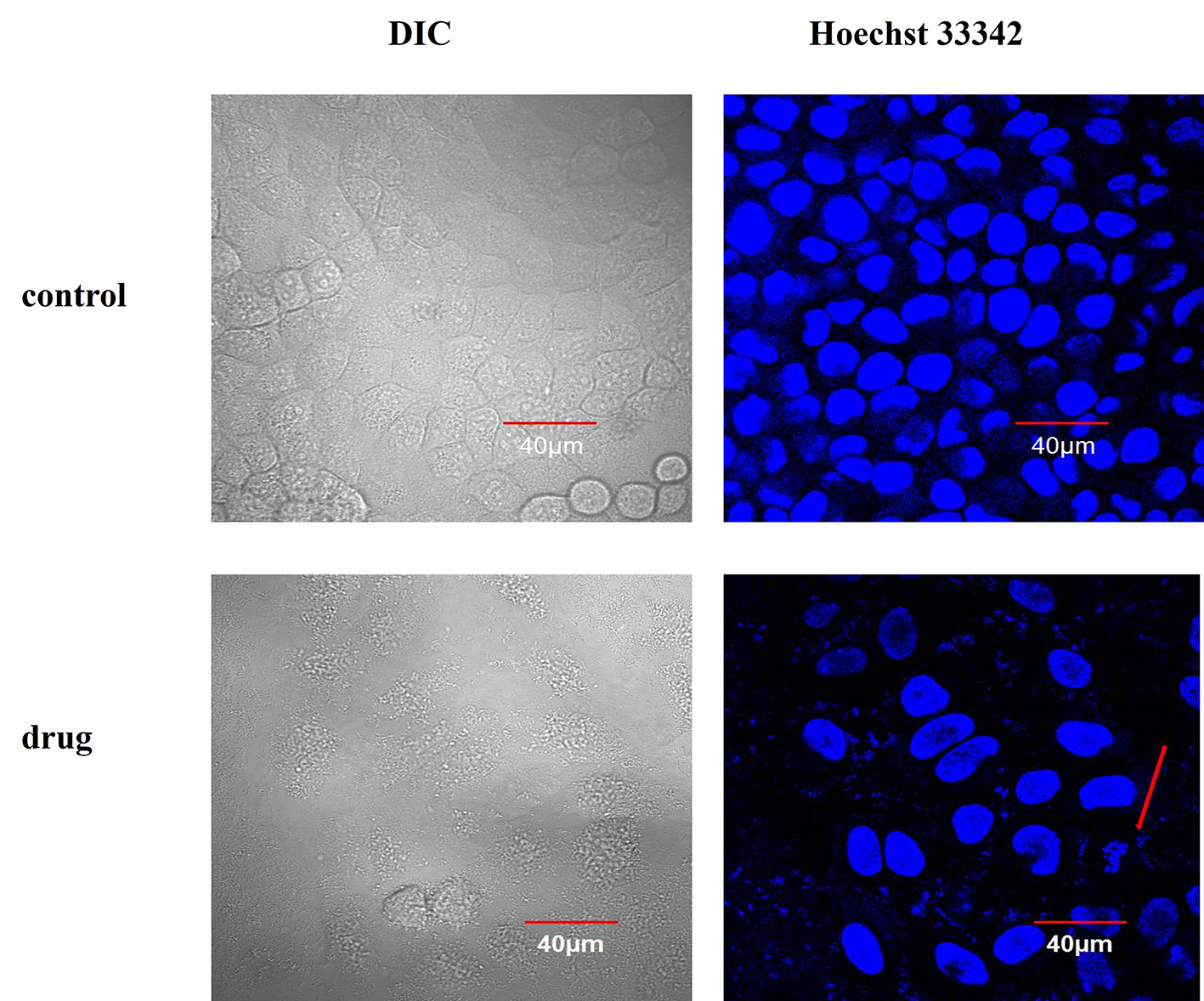

Supplement: S4 Fig — The corresponding POM concentration was 80 μg/mL. Scale bar in the CLSM is 40 μm (Original magnification 60×). Arrow shows apoptotic nuclear. (TIF) [file pone.0181018.s004.tif]
